# Management of Ulnar Nerve Tuberculoma: A Challenge to Plastic Surgeons

**DOI:** 10.7759/cureus.23872

**Published:** 2022-04-06

**Authors:** Rajesh Maurya, Mohd Altaf Mir, Prasenjit Singh

**Affiliations:** 1 Plastic Surgery, All India Institute of Medical Sciences, Bathinda, IND; 2 Plastic Surgery, All India Institute of Medical Sciences, Bhatinda, IND

**Keywords:** caseous necrosis, claw-hand, antitubercular therapy, ulnar nerve, tuberculoma

## Abstract

Tuberculoma is a well-circumscribed, space-occupying lesion. However, the involvement of peripheral nerves is rare. Here, we report a rare case of left ulnar nerve tuberculoma managed by surgical exploration and tangential excision of tuberculoma lesion sparing, most of the nerve fibers followed by antitubercular treatment. The patient responded well to the management strategy with improvement in motor and sensory activity of the affected limb.

## Introduction

Tuberculoma is a well-circumscribed, space-occupying lesion [1,2] and tuberculoma involving the peripheral nervous system is rare. Pathogenesis of tuberculoma spreading to peripheral nerves is unclear [1-3]. Peripheral nerve involvement usually occurs by direct infiltration from a tubercular lesion involving bone, joints, or lymph nodes [1-3]. The tuberculoma arises from tubercular foci and starts as a non-caseating granuloma characterized by central multi-nucleated giant cells surrounded by epithelioid cells without central necrosis, but in later stages progresses into caseating granuloma.

Here, we report a rare case of ulnar nerve tuberculoma managed by surgical exploration, tangential excision of the lesion followed by antitubercular treatment with excellent postoperative results.

## Case presentation

History and Examination

**Figure 1:**
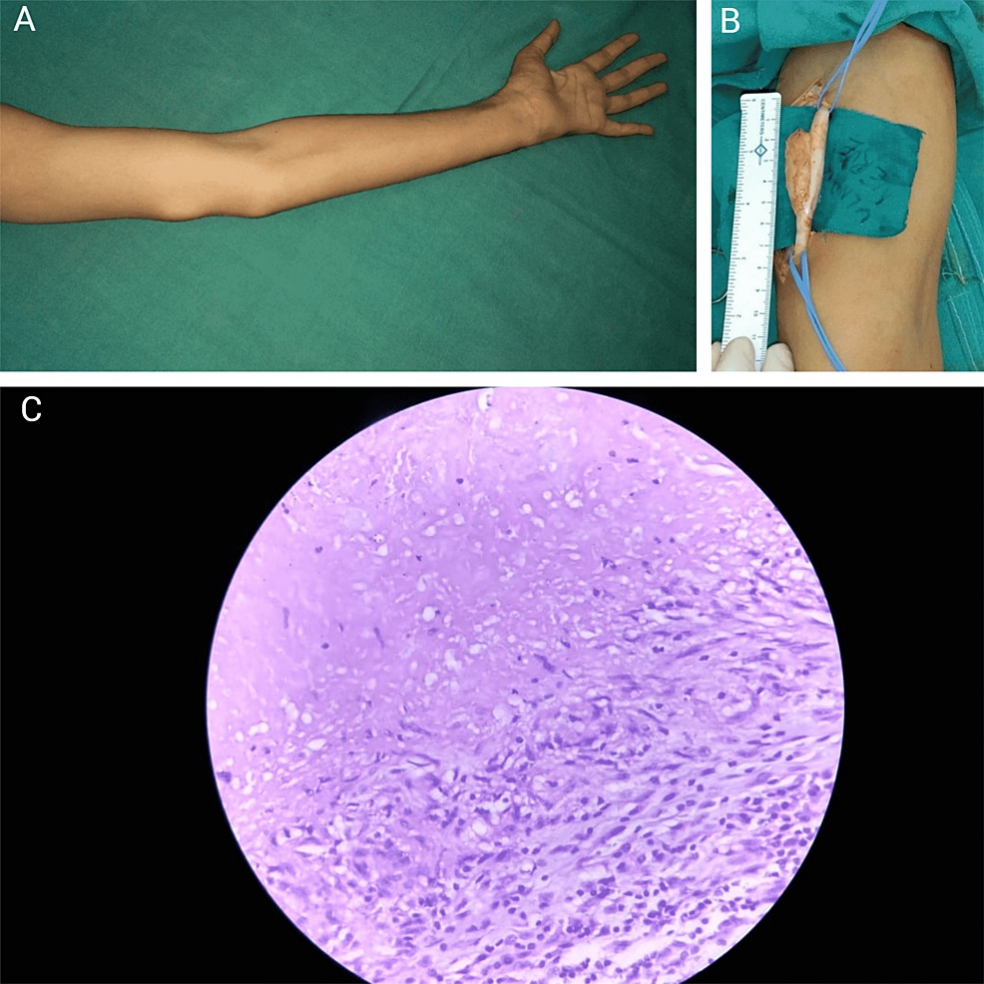
Characteristics of the lesion. Panel A depicts the swelling just proximal to the medial epicondyle of the left humerus and the wasting of hypothenar muscles on the affected side. Panel B is an intraoperative photograph of the ulnar nerve and the tuberculoma involving less than half of the circumference of the nerve. Panel C is a microphotograph depicting fibrous tissue, epithelioid cells, caseous necrosis, and Langerhans giant cells with granulomas suggesting the diagnosis of tuberculosis.

Eighteen years old male presented with 9-months history of progressive swelling in the left arm, tingling sensation, weakness and numbness in their left hand and forearm. There was no history of trauma or contact with the infected individual or material. On examination, there was a well-defined and mobile swelling with 3cm of size over the medial aspect of the left arm just proximal to the elbow joint (figure 1A). Neurological examination showed loss of sensation and the distribution of ulnar nerve and wasting of hypothenar muscles on the affected side. No lymphadenopathy was found in the axilla, neck, and other regions in clinical examination. The pulmonary radiograph did not show any feature suggestive of pulmonary tuberculosis.

Magnetic resonance imaging of the left arm and forearm, including the elbow joint, was performed. A heterogeneous, well-defined altered signal intensity lesion was seen involving a distal third of the medial aspect of the left arm in the subcutaneous and intramuscular plane, 3.3cm above the medial epicondyle of the humerus and eccentrically located within the humerus the ulnar nerve, displacing the nerve fascicles laterally with mild thickening of the nerve. The lesion was relatively encapsulated without any extra neural extension. In the nerve conduction test, the compound muscle action potential of the left ulnar nerve showed lower amplitude on stimulation.

Operation

Under the brachial block, the lesion was explored. A medial longitudinal incision was made, and the nerve sheath was opened. Granular caseous material was seen enclosed in a fibrous capsule along the course of a nerve, involving the full thickness of the nerve (fig 1B). The entire lesion and the involved nerve measured about 5cm long. Lesion excised in toto, sparing the 75% uninvolved nerve fibers. The excised specimen was explored, some caseous material was removed for microbiological assessment, and the rest of the specimen was sent for histopathological examination. The wound was washed and irrigated thoroughly with normal saline. The wound was closed after the placement of a closed suction drain. The hand was splinted in a functional hand position.

Histopathological and microbiological examination

Histopathological examination of the specimen showed fibrous tissue, epithelioid cells, caseous necrosis, and Langerhans giant cells with granulomas, suggesting tuberculosis diagnosis (fig 1C). Acid-fast bacilli were seen in Ziehl Nelsen staining, and micro-bacterium Bovis was confirmed on BACTEC rapid culture.

Postoperative Course

**Figure 2:**
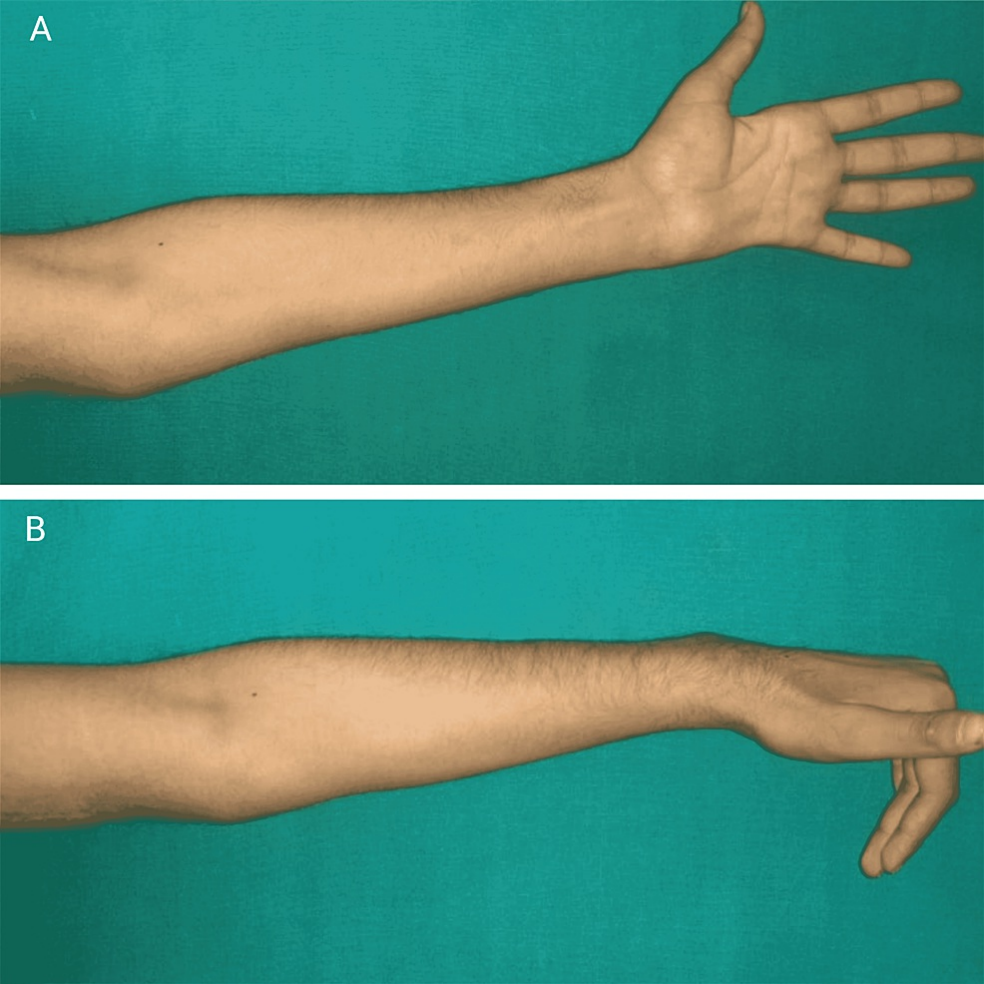
Postoperative clinical photographs at 18 months. The improvement in the movement of little and ring fingers, extension as depicted in (fig 2A), and flexion (fig 2B).

The patient was followed up in the postoperative period. The antitubercular treatment was started, and there was a significant improvement in his hand's motor and sensory activity at 18 months of follow-up. The power of the left-hand grip had also improved. There was a significant improvement in two-pint discrimination at the fingertips of little and ring fingers and improvement in the movement of little and ring fingers, extension as depicted in (fig 2A), and flexion (fig 2B).

## Discussion

In our patient, the histopathology report confirmed the presence of granuloma, epithelioid cells, and caseous necrosis. However, no other signs and symptoms of tuberculosis apart from the ulnar nerve involvement were present. Hence, it ruled out a hematogenous spread in our patient.

Nucci et al. [1] and Sinha [2] reported ulnar nerve tuberculosis in which tuberculosis foci were absent. According to Prakashvn SA [3], tuberculous bacilli is bloodborne. No typical symptoms of tuberculosis were present in our patient with ulnar nerve tuberculoma. Only the ulnar nerve innervated area showed weakness and atrophy of muscles, paresthesia along the ring and little finger, and ulnar palm side. Chandra et al. [4] also reported a 7-year-old girl having ulnar nerve tuberculoma in the right arm with a 1-year history of weakness in her right hand and forearm. Song et al. [5] also described a case of ulnar nerve tuberculoma over the ulnar aspect in a 25-year-old patient with a chief complaint of numbness on his left hand and forearm with no history of trauma or contact with any individual having active tuberculosis.

The basic method tried in our case was shave excision of the lesion longitudinally parallel to the direction of nerve fibers (tangential excision), sparing the uninvolved nerve fibers, followed by antitubercular therapy. In combination with antitubercular therapy, the above procedure yielded significant improvement in motor and sensory activity.

## Conclusions

Early exploration of the infected tissues combined with antitubercular multidrug therapy gives good functional results and prevents recurrence. The management of nerve granuloma involving less than half of diameter may be managed with antitubercular multidrug therapy. However, surgical exploration and medical management are the keys to management when involvement is more.
